# Photodegradation of the phenylpyrazole insecticide ethiprole in aquatic environments and a comparison with fipronil

**DOI:** 10.1007/s11356-024-34767-9

**Published:** 2024-08-27

**Authors:** Soichiro Hirashima, Tomoko Amimoto, Yoko Iwamoto, Kazuhiko Takeda

**Affiliations:** 1 Seto Inland Sea Carbon-Neutral Research Center, Hiroshima University, 1-7-1 Kagamiyama, Higashi, Hiroshima, 739–8521 Japan; 2 Natural Science Center for Basic Research and Development, Hiroshima University, 1-3-1 Kagamiyama, Higashi, Hiroshima, 739–8526 Japan; 3 Graduate School of Integrated Sciences for Life, Hiroshima University, 1-7-1 Kagamiyama, Higashi, Hiroshima, 739–8521 Japan; 4 Faculty of Integrated Arts and Sciences, Hiroshima University, 1-7-1 Kagamiyama, Higashi, Hiroshima, 739–8521 Japan

**Keywords:** Pesticide, Photostability, Photo-dechlorination products, High-resolution mass spectrometry, LC–MS/MS

## Abstract

Ethiprole (ETH) is a phenylpyrazole insecticide that is used worldwide as an alternative to fipronil (FIP). Research on the photodegradation of ETH in aquatic environments has been limited compared with that on FIP. In this study, to clarify the photodegradation of ETH in aquatic systems, the photodegradation pathway and products were investigated using liquid chromatography and liquid chromatography-tandem mass spectrometry. We also determined the photochemical half-lives (*t*_1/2_) of ETH and its main degradation products. The primary photodegradation pathway was cyclization/dechlorination and hydroxylation/dechlorination of ETH to form the didechlorinated products (benzimidazole of des-chloro-hydroxy-ETH). Some newly identified photodegradation products and analogs of FIP photodegradation products were also detected as minor products. We compared the photodegradation of ETH with that of FIP under the same conditions. Didechlorinated products of ETH and FIP had the highest photostability. However, although the photochemical *t*_1/2_ of EHT was 2.7 times that of FIP, the photochemical *t*_1/2_ of the didechlorinated product of ETH was approximately one-third that of the didechlorinated product of FIP. This comparison of the photochemical processes of ETH and FIP provides new insight into the persistence and characteristics of both insecticides in the environment.

**Supplementary Information:**

The online version contains supplementary material available at 10.1007/s11356-024-34767-9.

## Introduction

Ethiprole (5-amino-1-[2,6-dichloro-4-(trifluoromethyl)phenyl]-4-(ethylsulfinyl)-1*H*-pyrazole-3-carbonitrile; ETH) is a new type of phenylpyrazole insecticide that is used as an alternative to fipronil (5-amino-1-[2,6-dichloro-4-(trifluoromethyl)phenyl]-4-[(trifluoromethyl)sulfinyl]-1*H*-pyrazole-3-carbonitrile; FIP) (USEPA (United States Environmental Protection Agency), 2011; FSCJ (Food Safety Commission of Japan), 2014; JMPR (Joint FAO/WHO Meeting on Pesticide Residues), 2018). ETH and FIP function as a γ-aminobutyric-acid–dependent chloride ion channel inhibitors in insects, and cause death by destroying the central nervous system (Cole et al. 1993; Bobé et al. 1998; Gant et al. 1998; Aajoud et al. 2003; Caboni et al. 2003; Zhao et al. 2010). EHT is less toxic than FIP to non-target organisms, such as mammals (Caboni et al. 2003). In China, FIP was banned for agricultural use in 2009 because of its high toxicity, and ETH has since been used as an alternative pesticide (Sheng et al. 2018; Li et al. 2019). In Japan, domestic shipment of ETH has increased since 2005 and reached 35.6 tons in 2021, while domestic shipment of FIP has decreased since 2010 (Fig. S1) (National Institute for Environmental Studies WebKis-Plus system; http://w-chemdb.nies.go.jp/).

Although the substitution of FIP with ETH has progressed, research on the environmental risks of ETH has been limited. A number of degradation processes have been reported for FIP, including biodegradation, oxidation, reduction, hydrolysis, and photodegradation, among which photochemical degradation is known to be the main degradation pathway in aquatic environments (Singh et al. 2021). Compared with FIP, degradation products of FIP have similar or greater toxicity (Hainzl et al. 1998; Gunasekara et al. 2007; Singh et al. 2021). FIP and its degradation products are increasingly being found to persist in rivers and coastal waters worldwide (Hano et al. 2019; Li et al. 2019; Pan et al. 2020; He et al. 2021; Naumann et al. 2022; Onduka et al. 2022; Sutton et al. 2022; Shi et al. 2023; Uchida et al. 2023; Xiong et al. 2023). Further elucidation of their environmental dynamics is conducted because they are contaminants of emerging concern (Sutton et al. 2022). For FIP, FIP-desulfinyl has been reported as the main photochemical product and its photostability is roughly 20 times that of FIP (Ngim and Crosby 2001; Hirashima et al. 2023). Furthermore, it is more toxic than FIP (Hainzl et al. 1998; Gunasekara et al. 2007). Recently, we found a novel photodegradation pathway of FIP-desulfinyl to another photostable product (Hirashima et al. 2023). The main photodegradation pathways of ETH described in previous studies are cyclization/dechlorination and hydroxylation/dechlorination (USEPA (United States Environmental Protection Agency), 2011; FSCJ (Food Safety Commission of Japan), 2014; JMPR (Joint FAO/WHO Meeting on Pesticide Residues), 2018; Chen et al 2019). On the other hand, studies on the degradation products of ETH have been very limited and it is unclear how stable the degradation products are compared with those of FIP.

The aim of this study was to understand ETH photodegradation in aquatic systems. We investigated the photochemical products and kinetics of ETH using liquid chromatography (LC) and LC-tandem mass spectrometry (LC–MS/MS) with an Orbitrap instrument. The photochemical *t*_1/2_ of the main photodegradation products under natural sunlight were estimated. We compared the photochemical *t*_1/2_ of ETH and its main photodegradation products with those for FIP and its main photodegradation products from a previous study conducted under the same experimental conditions (Hirashima et al. 2023). This is the first study to compare the photochemical degradation of ETH and FIP in aqueous systems. The results provide useful insight for the development of new phenylpyrazole insecticides with low environmental risk.

## Material and methods

### Reagents and water

ETH (99.4% pure) was obtained from Fujifilm Wako Pure Chemical Corp. (Osaka, Japan) and used without further purification. Methanol (MeOH), acetonitrile (MeCN), and all other chemicals were of reagent grade, LC grade, or better and used without purification. Milli-Q water (18.2 MΩ cm) was prepared using a Milli-Q Elix UV-5 and a Milli-Q Advantage system (Millipore, Tokyo, Japan).

### Photodegradation experiments

ETH photodegradation experiments were performed using a solution of 2.5 mg/L ETH in 2.5% (v/v) aqueous MeOH using the conditions described by Hirashima et al. (2023). The ETH solution was clear and did not contain any precipitation. After storage of the ETH solution in flat-bottomed quartz bottles in the dark for 360 min, the LC analysis peak intensity did not change (Table S1) and no new peaks for degradation products appeared. Therefore, no precipitation, adsorption, or degradation of ETH would occur during the experiment.

For the photodegradation experiments, a custom-made photochemical experimental system with a 500 W ozone-free xenon lamp (SX-UI501HQ; Ushio, Tokyo, Japan) and an optical long-pass filter with a cut-on wavelength of 309 nm (UV N-WG305; Edmund Optics, Tokyo, Japan) was used as a solar simulator (Fig. S2, Hirashima et al. 2023). A 50-mL aliquot of ETH solution in a flat-bottomed quartz bottle was irradiated at 20 °C ± 2 °C. The photodegradation experiments were conducted for up to 360 min, during which the formation and degradation of the two main photodegradation products from ETH were observed. ETH and its photodegradation products were analyzed using an isocratic reversed-phase LC system and a LC–MS/MS system, as described below.

The light intensity of the solar simulator was determined using chemical actinometry from the photodegradation rate of 8 μM 2-nitrobenzaldehyde (2NB) irradiated using the same photochemical system for ETH (Takeda et al. 2014, 2017). The concentration of 2NB was determined using an isocratic reversed-phase LC system. The 2NB photodegradation rate during the experiment ranged from 0.0389 to 0.0411 s^−1^ (average: 0.0397 s^−1^). The 2NB photodegradation rate under natural sunlight on 7 October 2002 in Higashi-Hiroshima at noon under a clear sky was 0.0093 s^−1^, which showed that the light intensity we used in this study was 4.27 times that of natural sunlight at noon under a clear sky (Takeda et al. 2014, 2017). A ratio of 0.32 was used for the ratio of the estimated 24-h average radiant intensity to the maximum intensity at noon (light intensity coefficient to account for diurnal variations in solar radiation intensity, details were mentioned in the previous report by Takeda et al. (2014)).

To detect minor photodegradation products as well as major products, some samples were preconcentrated by solid-phase extraction (SPE) before LC–MS/MS. The Sep-Pak Plus PS-2 cartridge (Waters, Tokyo, Japan) was used as SPE cartridges. For these samples, 30 mL was concentrated using a SPE cartridge that was activated and preconditioned with 5 mL of MeOH and 2.5% aqueous MeOH. After washing the SPE cartridge with 5 mL of 2.5% aqueous MeOH, the analytes were eluted using 1.5 mL of MeOH.

### Analysis by LC and LC–MS/MS

The time profiles of ETH and the main photodegradation products during the photodegradation experiments were determined by isocratic LC 10Ai system with an ultraviolet (UV) detector (Shimadzu, Kyoto, Japan). Separation was achieved using a reversed-phase C18 column (Cosmosil 5C18-MS-II, 150 × 4.6 mm i.d., 5 μm particle size; Nacalai Tesque, Kyoto, Japan). The mobile phase was a mixture of Milli-Q water, MeCN, MeOH, and formic acid in a volume ratio of 45:30:25:0.1 with a flow rate of 1.0 mL min^−1^. The column oven was maintained at 35 °C and the UV detector wavelength was 280 nm. The sample was injected via an injection valve with a 20 μL-sample loop. For the determination of 2NB in chemical actinometry, the same LC system was used but the mobile phase was a 45:55 (v/v) mixture of Milli-Q water and MeCN and the UV detector wavelength was 260 nm.

The photodegradation products of ETH were identified by LC–MS/MS with an LTQ Orbitrap XL high-resolution MS/MS system (Thermo Fisher Scientific) coupled with a Vanquish Flex UHPLC system (Thermo Fisher Scientific, Tokyo, Japan). Mass spectra were obtained using positive and negative mode electrospray ionization and were analyzed using Xcalibur QUAL Browser software (Thermo Fisher Scientific) (Nagasaka et al. 2017).

### Simulation of temporal changes in the ETH and degradation product concentrations using a sequential degradation model

The time courses of photodegradation of ETH and two main degradation products were simulated using a sequential degradation model, and the degradation rate constants (*k*) of the compounds were estimated. In the model, four compounds (*A*, *B*, *C*, and *D*) were sequentially photodegraded by first-order kinetics without any side reactions or back reactions as shown in Eq. 1 (Hirashima et al. 2023).

1 $$A \stackrel{{k}_{a}}{\to } B \stackrel{{k}_{b}}{\to } C \stackrel{{k}_{c}}{\to } D,$$

where *k*_*a*_, *k*_*b*_, and *k*_*c*_ are the rate constants for first-order kinetics. The rate laws are shown below.

2 $$\frac{d\left[A\right]}{dt}={-k}_{a}[A],$$

3 $$\frac{d\left[B\right]}{dt}={k}_{a}\left[A\right]-{k}_{b}\left[B\right],$$

4 $$\frac{d\left[C\right]}{dt}={k}_{b}\left[B\right]-{k}_{c}\left[C\right],$$

5 $$\frac{d\left[D\right]}{dt}={k}_{c}\left[C\right].$$

The equations were solved by finding a general solution for the homogeneous differential equation and particular solutions for the non-homogeneous equations. The concentrations of the compounds were calculated using the general solutions shown below.

6 $$\left[A\right]={A}_{0}{\text{e}}^{-{k}_{a}t},$$

7 $$\left[B\right]={A}_{0}\frac{{k}_{a}}{{k}_{b}-{k}_{a}}\left\{{\text{e}}^{-{k}_{a}t}-{\text{e}}^{-{k}_{b}t}\right\},$$

8 $$\left[C\right]={A}_{0}\frac{{k}_{a}{k}_{b}}{{k}_{b}-{k}_{a}}{\text{e}}^{-{k}_{c}t}\left\{\frac{1}{{k}_{c}-{k}_{a}}\left({\text{e}}^{\left({k}_{c}-{k}_{a}\right)t}-1\right)-\frac{1}{{k}_{c}-{k}_{b}}\left({\text{e}}^{\left({k}_{c}-{k}_{b}\right)t}-1\right)\right\},$$

9 $$\left[D\right]={A}_{0}-\left[A\right]-\left[B\right]-\left[C\right],$$

where *A*_0_ is the initial concentration of *A*. We assumed that the initial concentration of *A* was 1 (*A*_0_ = 1) and that the initial concentrations of *B*, *C*, and *D* at *t* = 0 were zero. The *t*_1/2_ was calculated as follows:

10 $${t}_{1/2}=\frac{ln(2)}{k}.$$

## Results and discussion

### Photodegradation of ETH and chemical structures of the main degradation products

**Fig. 1:**
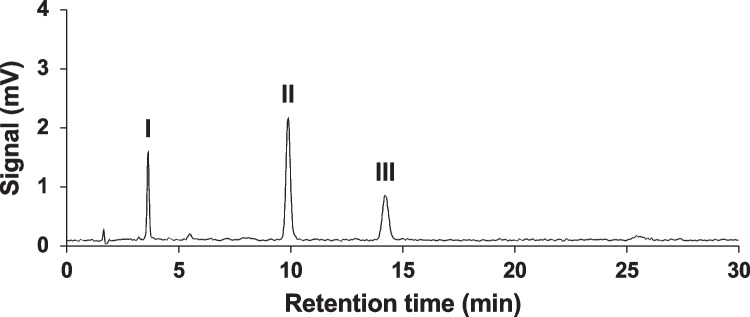
A UV chromatogram of 2.5 mg/L ETH in 2.5% aqueous MeOH irradiated under a solar simulator for 30 min. The UV detection wavelength was 280 nm

**Fig. 2:**
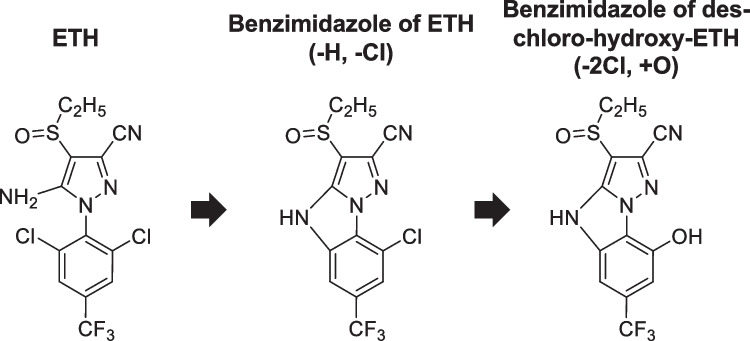
Main photodegradation process of ETH and chemical structures of the degradation products

A UV chromatogram of 2.5 mg/L ETH in 2.5% aqueous MeOH irradiated under the solar simulator for 30 min is shown in Fig. 1. Other representative chromatograms with different irradiation times are shown in Fig. S3. Three major peaks (I–III) were found in the chromatogram acquired using a UV detection at 280 nm. The analytical data of LC–MS/MS was used to identify the chemical structures of these three peaks. The *m/z* ratios of the deprotonated molecules ([M-H]^−^) and the major MS/MS fragments for the peaks are listed in Table 1, and the estimated chemical structures are shown in Fig. 2. Peak II was confirmed as ETH using the retention time and *m/z* ratio of the standard solution of ETH. From the *m/z* ratio of [M-H]^−^, peak I was identified as the benzimidazole of des-chloro-hydroxy-ETH, and peak III was identified as the benzimidazole of ETH. The MS spectra of the benzimidazole of ETH and benzimidazole of des-chloro-hydroxy-ETH showed characteristic isotope patterns for the chlorine atoms (Fig. S4). There are no previous reports giving the *m/z* ratios of MS/MS fragments of the benzimidazole of ETH and benzimidazole of des-chloro-hydroxy-ETH. A similar desorption pattern was observed for the MS/MS fragments of benzimidazole of ETH and benzimidazole of des-chloro-hydroxy-ETH. This was for a fragment ion formed by the loss of two hydrogen atoms and a cyano group, or two nitrogen atoms in the pyrazole ring from [M-H]^−^. The three major peaks were also detected in electrospray ionization positive mode and the *m/z* ratios for protonated molecules ([M-H]^+^) were consistent with the chemical structures in Fig. 2 (Table 1).

**Table 1:** LC–MS/MS results for the chromatogram in Fig. 1. The MS/MS (*m/z*) ratios are shown in order of peak intensity

	ESI^−^		ESI^+^	
Compound	MS (*m/z*)	MS/MS (*m/z*)	MS (*m/z*)	Reference
ETH	394.98	331.0 (-3H, -Cl, -CN), 359.0 (-H, -Cl)	396.99	Standard
Benzimidazole of ETH	359.00	331.0 (-2H, -CN, or -N–N)	361.01	JMPR (Joint FAO/WHO Meeting on Pesticide Residues), 2018
Benzimidazole of des-chloro-hydroxy-ETH	341.03	313.0 (-2H, -CN, or -N–N)	343.05	JMPR (Joint FAO/WHO Meeting on Pesticide Residues), 2018

The results show that cyclization/dechlorination of ETH formed a benzimidazole of ETH, followed by hydroxylation/dechlorination to form a benzimidazole of des-chloro-hydroxy-ETH. The temporal changes of these three compounds during photodegradation in the present study (discussed later) were consistent with this pathway. The main photodegradation pathway in this study agrees with a previous study (JMPR (Joint FAO/WHO Meeting on Pesticide Residues), 2018).

### Chemical structures of the minor products and ETH degradation pathways

**Fig. 3:**
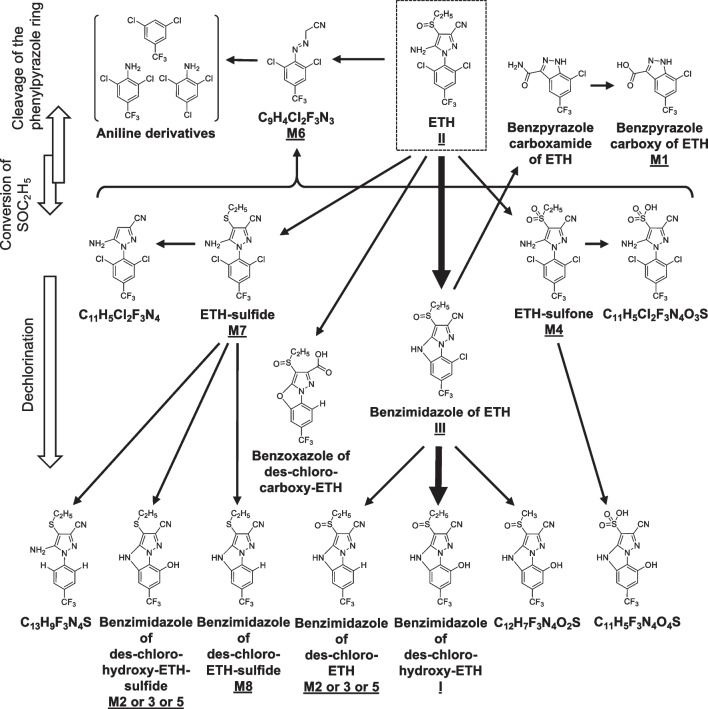
Photodegradation pathways for ETH in aquatic environments. Paths indicated by bold arrows are main photodegradation processes

In the total ion chromatogram of the precursor ions obtained by LC–MS/MS (total ion chromatogram of LC–MS), many other peaks (M1–M5, M7, and M8 in Fig. S5) appeared in addition to the three main photodegradation products. The *m/z* ratios for the MS and MS/MS of these peaks are summarized in Table S2, and the photodegradation pathways for ETH based on this study and previous works are shown in Fig. 3. The estimated chemical structures of the compounds for peaks M1, M4, and M7 have been reported elsewhere (USEPA (United States Environmental Protection Agency), 2011; FSCJ (Food Safety Commission of Japan), 2014; JMPR (Joint FAO/WHO Meeting on Pesticide Residues), 2018; Chen et al 2019). The *m/z* ratios and chemical structures of the compounds for peaks M2, M3, M5, and M8 have not been reported previously. Peaks M2, M3, and M5 had the same *m/z* ratio (*m/z* = 325.04, C_13_H_9_F_3_N_4_OS) and could be produced by two possible isomers: a benzimidazole of des-chloro-ETH, and a benzimidazole of des-chloro-hydroxyl-ETH-sulfide. Further information for the three peaks was not obtained in this study. The estimated chemical structure of the compound for peak M8 was a benzimidazole of des-chloro-EHT-sulfide (Fig. 3). The peak M6 (*m/z* = 279.97, C_9_H_4_Cl_2_F_3_N_3_) observed in the extracted ion chromatogram was attributed to a non-dechlorinated product, (2,6-dichloro-4-trifluoromethyl-phenylazo)-acetonitrile, which was reported in the photodegradation of ETH by Chen et al. (2019) and in that of FIP by Hirashima et al. (2023) (Figs. S6 and S7, and Table S2). The photodegradation pathway after M6 was estimated with reference to the photodegradation pathway of FIP (Raveton et al. 2006).

The ETH photodegradation pathways, including newly detected compounds in this study and previously reported compounds (USEPA (United States Environmental Protection Agency), 2011; FSCJ (Food Safety Commission of Japan), 2014; JMPR (Joint FAO/WHO Meeting on Pesticide Residues), 2018; Chen et al 2019), are summarized in Fig. 3. The main photodegradation pathway of ETH was cyclization/dechlorination and hydroxylation/dechlorination reactions maintaining sulfinyl structure, unlike the case of FIP (Hirashima et al. 2023). The main photodegradation pathway of ETH did not include desulfinylation reactions.

According to the photodegradation pathway of FIP (Singh et al. 2021; Hirashima et al. 2023), the benzimidazole of des-chloro-hydroxyl-EHT-sulfide (*m/z* = 325.04, M2 or 3 or 5) in Fig. 3 was thought to be formed from ETH-sulfide (M7) by cyclization/dechlorination and hydroxylation/dechlorination reactions. However, the benzimidazole of des-chloro-hydroxyl-EHT-sulfide (M2 or 3 or 5) could form by reduction of the benzimidazole of des-chloro-hydroxyl-EHT (I), which is the primary photo-didechlorinated product of ETH. Further research is needed to understand the photodegradation process of ETH.

### Temporal changes in the concentrations of the main photodegradation products and the degradation kinetics

**Fig. 4:**
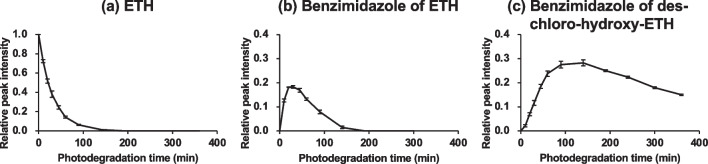
Temporal profiles of the main products of ETH photodegradation determined by LC (*n* = 3). The relative peak intensity indicates the ratio between the peak area at photodegradation time *t* and the initial ETH peak area (*t* = 0)

Temporal changes for the three main degradation products are shown in Fig. 4. The concentrations of these compounds are expressed as peak intensities relative to the initial ETH peak area (ratio between the peak area at photodegradation time *t* and the initial peak area of ETH). The temporal changes supported a sequential photodegradation pathway from ETH to the benzimidazole of des-chloro-hydroxy-ETH via the benzimidazole of ETH.

**Fig. 5:**
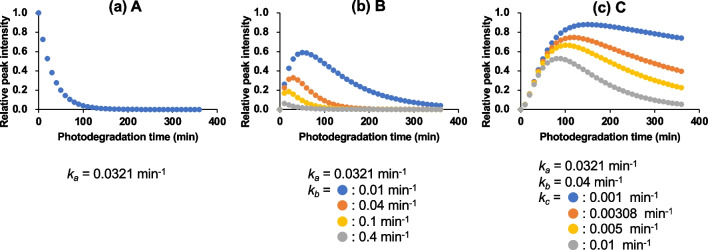
Simulations of the temporal profiles by the sequential degradation model

To assess the photodegradation of ETH and its photodegradation products, a simple first-order kinetics model and sequential degradation model based on that of Hirashima et al. (2023) were used to estimate the *k* and *t*_1/2_ of the degradation products. The degradation rate for ETH (*k*_*a*_) was estimated simply from the degradation profile of ETH between 0 and 60 min using Eq. 6. The degradation rate for benzimidazole of des-chloro-hydroxy-ETH (*k*_*c*_) was also estimated from its time profile during 190–360 min, in which peak intensities of both ETH and benzimidazole of ETH were negligible small, using a simple first-order kinetics model. For the benzimidazole of ETH, a sequential degradation mode, in which the compounds were photochemically decomposed sequentially without side reactions and without back reactions, was applied to estimate the degradation rate (*k*_*b*_). The temporal profile simulation results are shown in Fig. 5. The temporal profile for the benzimidazole of ETH was calculated using several reaction rate constants *k*_*b*_. The temporal profile simulated with *k*_*b*_ = 0.04 min^−1^ reproduced the observed profile (Fig. 5).

The *k* and *t*_1/2_ under the solar simulator and natural sunlight of a clear sky at noon are summarized in Table 2. The predicted *t*_1/2_ values under natural sunlight were 1.54 h for ETH, 1.23 h for the benzimidazole of ETH, and 16.0 h for the benzimidazole of des-chloro-hydroxy-ETH. The *t*_1/2_ of the benzimidazole of des-chloro-hydroxy-ETH under natural sunlight including diurnal changes was 2.1 days, which was calculated by dividing the predicted *t*_1/2_ by the light intensity factor (0.32). The persistence of this didechlorination product of ETH was about 10 times that of ETH and the benzimidazole of ETH. Therefore, the benzimidazole of des-chloro-hydroxy-ETH is expected to accumulate in aquatic environments because of its photostability. Furthermore, it could be an important indicator of long-term ETH contamination in aquatic environments. Therefore, the toxicities and long-term risks of the benzimidazole of des-chloro-hydroxy-ETH, and of ETH and its derivatives, to aquatic organisms and the environment need to be carefully evaluated.

**Table 2:** Photodegradation rate constants (*k*) and half-lives (*t*_1/2_) for ETH and its photodegradation products under a solar simulator and natural sunlight from a clear sky at noon

	Under solar simulator		Under natural sunlight
Compound	*k* (min^−1^)	*t*_1/2_ (min)	*t*_1/2_ (h)
ETH	0.0321	21.6	1.54
Benzimidazole of ETH	0.04	17.3	1.23
Benzimidazole of des-chloro-hydroxy-ETH	0.00308	225	16.0

### Comparison of the photochemical degradation of FIP and ETH

In this study, the photochemical experiments were conducted using the same experimental setup and conditions as those used in the photodegradation of FIP reported previously (Hirashima et al. 2023). So, we compared the photochemical degradation rate constants of FIP and ETH. The photochemical half-lives (*t*_1/2_) of FIP and ETH under natural sunlight determined in this study are summarized in Table 3. The *t*_1/2_ of ETH in this study was 2.7 times that of FIP in our previous study under the same conditions (Hirashima et al. 2023). According to the *Pesticide Evaluation Report* (FSCJ (Food Safety Commission of Japan), 2014; FSCJ (Food Safety Commission of Japan), 2016), the ETH *t*_1/2_ was 2.7 times that of FIP in sterile buffer (pH 5) and 1.5 times that of FIP in sterile natural water under a Tokyo spring sunlight equivalent (Table 3). Caboni et al. (2003) investigated the photodegradation of FIP and ETH in thin films on Petri dishes and found that the *t*_1/2_ of ETH was 2.3 times that of FIP. We can conclude that the photochemical stability of ETH is approximately 2–3 times that of FIP.

**Table 3:** Comparison of the photochemical half-lives (*t*_1/2_) of FIP and ETH under various conditions

Condition		*t*_1/2_ (h) of FIP	*t*_1/2_ (h) of ETH	Ratio of *t*_1/2_ (ETH/FIP)	Reference
Milli-Q water	Noon on a clear day in Higashi-Hiroshima City	0.578	1.54	2.7	FIP: Hirashima et al. 2023 ETH: This study
Sterile buffer (pH 5)	Spring sunlight in Tokyo	17.7	48.0	2.7	FIP: FSCJ (Food Safety Commission of Japan), 2016 ETH: FSCJ (Food Safety Commission of Japan), 2014
Sterile natural water	Spring sunlight in Tokyo	21.4	31.2	1.5	FIP: FSCJ (Food Safety Commission of Japan), 2016 ETH: FSCJ (Food Safety Commission of Japan), 2014
Thin films in Petri dishes	Exposed to direct sunlight in Berkeley, CA	6	14	2.3	Caboni et al. 2003

Many studies have reported FIP-desulfinyl as the main photodegradation product of FIP in aquatic systems (Hainzl and Casida 1996; Bobé et al. 1998; Ngim et al. 2000; Ngim and Crosby 2001; Raveton et al. 2006; Mianjy and Niknafs 2013). Hainzl and Casida (1996) reported that an amino or cyano group was required for desulfinylation to occur in the photodegradation of FIP. However, desulfinylation of ETH is not included in the main photodegradation pathway of ETH shown in this study (Fig. 3). Furthermore, the desulfinyl product of ETH in photoirradiation experiments has not been reported in previous studies (Caboni et al. 2003; USEPA (United States Environmental Protection Agency), 2011; FSCJ (Food Safety Commission of Japan), 2014; JMPR (Joint FAO/WHO Meeting on Pesticide Residues), 2018; Chen et al. 2019). From the above, desulfinylation seems to be an inherent reaction for FIP but not for ETH.

**Fig. 6:**
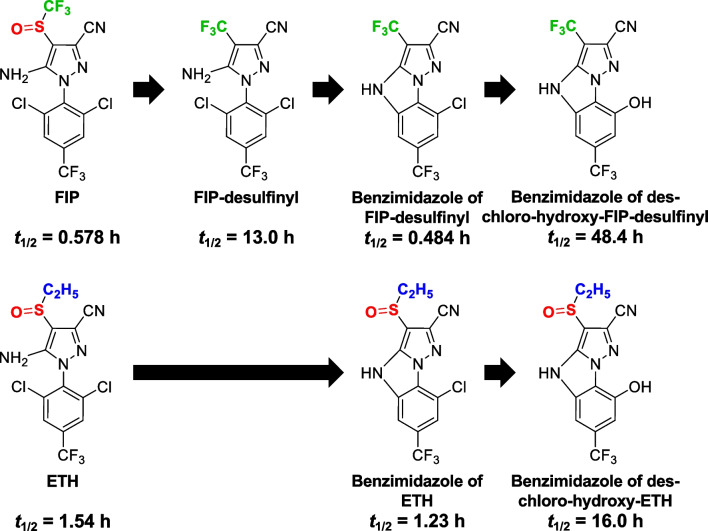
Comparison of the main photodegradation pathways between FIP and ETH. The photochemical half-lives (*t*_1/2_) under natural sunlight of FIP and ETH are also shown. The CF_3_ group (green) and the C_2_H_5_ group (blue) on the pyrazole ring are the specific substructures for FIP and ETH, respectively. The sulfinyl structure (SO, red) does not change in the main photodegradation pathway of ETH

Figure 6 compares the main photodegradation processes and *t*_1/2_ of ETH and FIP reported in this study and our previous study (Hirashima et al. 2023). As a common feature, the didechlorinated products had the highest photochemical stability for both FIP and ETH. Furthermore, the retention times of the didechlorinated products of FIP and ETH in reversed-phase LC analysis were shorter than those of the other degradation products, which suggested that they were more hydrophilic than the other degradation products of FIP and ETH. For both FIP and ETH, the photochemical end-products were hydrophilic and photostable didechlorinated compounds. Although parent ETH and FIP will be rapidly photochemically disappeared in natural environments, their cyclized and didechlorinated products were expectived to remain in aquatic environments. A comparison of main photodegradation pathways between FIP and ETH revealed a characteristic difference in the photochemical *t*_1/2_, as well as the desulfinylation. Although ETH was more photochemically stable than FIP, the *t*_1/2_ of the FIP photo-didechlorinated product (benzimidazole of des-chloro-hydroxy-FIP-desulfinyl reported in Hirashima et al. (2023)) was approximately three times that of the benzimidazole of des-chloro-hydroxy-ETH. FIP and ETH have similar chemical structures and main photodegradation reactions (other than desulfinylation) but their *t*_1/2_ reveal interesting differences, including in the photodegradation products.

### Risks of ETH and its photodegradation products in the aquatic environment

For FIP and FIP-desulfinyl, both environmental monitoring (Hano et al. 2019; Li et al. 2019; Pan et al. 2020; He et al. 2021; Naumann et al. 2022; Onduka et al. 2022; Sutton et al. 2022; Shi et al. 2023; Uchida et al. 2023; Xiong et al. 2023) and degradation processes (Singh et al. 2021; Hirashima et al. 2023) have been analyzed in various aquatic samples. ETH has been detected in multiple rivers and lakes at the same or higher concentrations than FIP (Kawasaki et al. 2018; Kiguchi et al. 2022). On the other hand, photodegradation products of ETH, such as benzimidazole of ETH and des-chloro-hydroxy-ETH, have not been reported in aquatic environments. Therefore, it is important to investigate the residual studies of ETH photodegradation products and their risks in comparison with those of FIP. The didechlorinated products of ETH and FIP, shown in Fig. 6, appear to be significant species for biological toxicity due to their photochemical stabilities. These didechlorinated products are expected to lose their original toxicity (neurotoxicity to insects) because the 2,6-dichloro structure on the benzene ring, which is considered essential for the insecticidal activity of phenylpyrazole insecticides, is lost (Ozoe et al. 2000; Ozoe 2004). On the other hand, the photochemical cyclization reactions have resulted in significant changes in the chemical structure, potentially leading to additional toxicity beyond that of the original compound. Detailed toxicity studies are needed on the didechlorinated products produced from FIP and ETH for both target and non-target organisms.

### Non-photochemical oxidation and reduction of ETH and its photodegradation products

For both FIP and ETH, oxidation and reduction of sulfinyl structures have been reported as key non-photodegradation reactions in nature, such as oxidation in organisms and reduction in anaerobic soil. (Brennan et al. 2009; FSCJ (Food Safety Commission of Japan), 2014; FSCJ (Food Safety Commission of Japan), 2016; JMPR (Joint FAO/WHO Meeting on Pesticide Residues), 2018; Singh et al. 2021). The oxidation or reduction products of FIP have been shown to persist in aquatic environments, and in some cases, these compounds have been detected at concentrations equal to or greater than those of FIP (Li et al. 2019; Pan et al. 2020; He et al. 2021; Naumann et al. 2022; Sutton et al. 2022; Shi et al. 2023; Uchida et al. 2023; Xiong et al. 2023). The main photodegradation pathway of ETH proceeds without desulfinylation, which is unlike that of FIP, suggesting that the main photodegradation products of ETH (i.e., benzimidazole of ETH and benzimidazole of des-chloro-hydroxy-ETH) retain the sulfinyl structure. Consequently, these compounds may undergo oxidation and reduction of the sulfinyl structure in natural environments similar to FIP and ETH (Fig. S8).

The oxidation and reduction of photo-dechlorination products to sulfinyl structures is presumed to be characteristic of the degradation of ETH compared with FIP. The results in this study suggested that the benzimidazole of des-chloro-hydroxyl-EHT-sulfide (*m/z* = 325.04, M2 or 3 or 5) could form by reduction of the benzimidazole of des-chloro-hydroxyl-EHT (I). However, the occurrence of ETH degradation products in aqueous environments has not been clarified. Furthermore, products of the oxidation and reduction of their sulfinyl structures have not been detected in rivers, coastal areas, or other natural waters. It is expected to elucidate the formation mechanism of ETH degradation products, including presumed degradation products, in natural water and to determine the conditions for their existence in aquatic environments.

## Conclusions

The main photodegradation pathway of ETH was identified as cyclization/dechlorination to a benzimidazole of ETH and hydroxylation/dechlorination to a benzimidazole of des-chloro-hydroxy-ETH. Some new minor photodegradation products were identified. No desulfinyl products of ETH were detected in the photodegradation products; however, ETH with an oxidized or reduced ethylsulfinyl group was observed as a minor photodegradation product. The *t*_1/2_ of the didechlorinated product of ETH (benzimidazole of des-chloro-hydroxy-ETH) was approximately 10 times that of ETH, which is the longest photochemical *t*_1/2_ among the main photodegradation products. Furthermore, the retention time of the benzimidazole of des-chloro-hydroxy-ETH in reversed-phase LC was shorter than those of the other degradation products, which suggested that it was more hydrophilic than ETH and the other degradation products. Therefore, the benzimidazole of des-chloro-hydroxy-ETH could remain at higher concentrations than ETH in the aquatic environment, as contaminants of emerging concern.

Recently, some newly developed phenylpyrazole insecticides have been investigated to optimize their insecticidal activity (Ozoe et al. 2000; Ozoe 2004; Jiang et al. 2014; Dong et al. 2023). In the development and commercialization of these new insecticides, it is necessary to assess their risks, including their toxicity towards non-target organisms, stability, and degradability in the environment.

## Supplementary Information

Below is the link to the electronic supplementary material.

Supplementary file1 (PDF 1604 KB)

## Data Availability

The datasets used and/or analyzed during the current study are available from the corresponding author on a reasonable request.
