# Supplementary material for: Photodegradation of the phenylpyrazole insecticide ethiprole in aquatic environments and a comparison with fipronil
Source: Environ Sci Pollut Res Int. 2024 Aug 27;31(40):53447–57. doi: 10.1007/s11356-024-34767-9 (PMC11379744; doi:10.1007/s11356-024-34767-9)
Supplement: Supplementary file 1 — Supplementary file1 (PDF 1604 KB) [file 11356_2024_34767_MOESM1_ESM.pdf]

SUPPORTING INFORMATION

Photodegradation of the phenylpyrazole insecticide ethiprole in aquatic environments and a comparison with fipronil

Soichiro Hirashima[1], Tomoko Amimoto[2], Yoko Iwamoto[1,3,4], Kazuhiko Takeda[1,3,4*]

1 Seto Inland Sea Carbon-neutral Research Center, Hiroshima University, 1-7-1 Kagamiyama, Higashi-Hiroshima 739–8521, Japan

2 Natural Science Center for Basic Research and Development, Hiroshima University, 1-3-1 Kagamiyama, Higashi-Hiroshima 739–8526, Japan

3 Graduate School of Integrated Sciences for Life, Hiroshima University, 1-7-1 Kagamiyama, Higashi-Hiroshima 739–8521, Japan

4 Faculty of Integrated Arts and Sciences, Hiroshima University, 1-7-1 Kagamiyama, Higashi-Hiroshima 739–8521, Japan

*Corresponding Author: Kazuhiko Takeda. E-mail: takedaq@hiroshima-u.ac.jp

1. Changes in Japanese domestic shipments of FIP and ETH

Fig. S1 Changes in Japanese domestic shipments of FIP and ETH (Based on the National Institute for Environmental Studies Web Kis-Plus system; http://w-chemdb.nies.go.jp/)

2. Stability of ETH in 2.5% MeOH aqueous solution

Table S1 Stability of ETH in Milli-Q water containing 2.5% MeOH

3. Appearance of the photodegradation equipment

Fig. S2 (a) Schematic diagram of photochemical experimental system
(b) Picture of the photodegradation equipment

4. Results of LC analysis

Fig. S3 UV chromatograms of aqueous solution of ETH containing 2.5% MeOH after (a) 0 min (before photoirradiation), (b) 30 min, (c) 90 min, and (d) 300 min photodegradation

5. Results of LC-MS/MS analysis

Fig. S4 Isotope pattern of (a) ETH, (b) Benzimidazole of ETH and (c) Benzimidazole of des-chloro-hydroxy-ETH in MS spectra

Fig. S5 The LC-MS total ion chromatogram (TIC) of photo-irradiated ETH solution after SPE concentration

(a) TIC of 30 min photo-irradiated solution after SPE concentration

(b) UV 280 nm chromatogram monitored simultaneously with (a)

(c) TIC of 300 min photo-irradiated solution after SPE concentration

(d) UV 280 nm chromatogram monitored simultaneously with (c)

Fig. S6 The LC-MS extracted ion chromatogram (EIC) (Mass range: *m/z* ratios = 279.96–279.98) of photo-irradiated ETH solution after SPE concentration

Upper: EIC of 0 min photo-irradiated solution after SPE concentration

Lower: EIC of 300 min photo-irradiated solution after SPE concentration

Fig. S7 Isotope pattern of MP6 in MS spectra

Table S2 LC-MS/MS results of minor products M1–M8 corresponding to TIC or EIC shown in Fig. S5 and S6. The MS/MS (*m/z*) ratios are shown in order of peak intensity

6. Photodegradation and non-photochemical oxidation and reduction of ETH

Fig. S8 Photodegradation and non-photochemical oxidation and reduction of ETH

1. Changes in Japanese domestic shipments of FIP and ETH

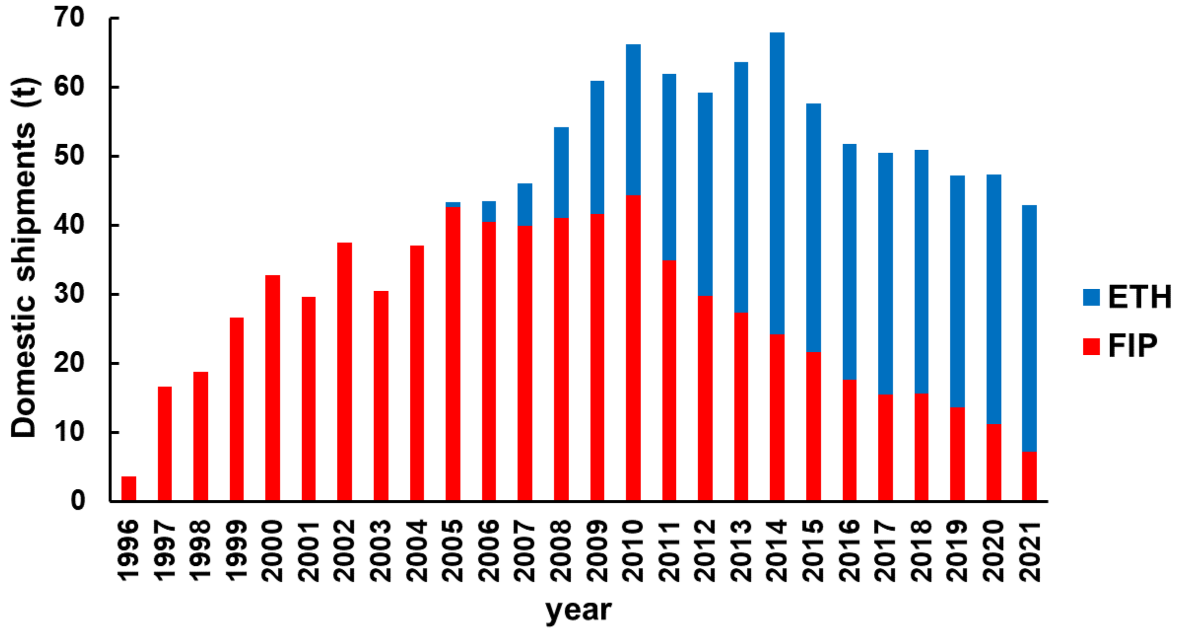

Fig. S1 Changes in Japanese domestic shipments of FIP and ETH (Based on the National Institute for Environmental Studies Web Kis-Plus system; http://w-chemdb.nies.go.jp/)

FIP domestic shipment was decreasing since 2010. On the other hand, domestic shipment of ETH has been increasing since 2005 and exceeded that of FIP as of 2013. The domestic shipment of ETH reached 35.6 tons in 2021.

2. Stability of ETH in 2.5% MeOH aqueous solution

Table S1 Stability of ETH in Milli-Q water containing 2.5% MeOH

	Relative peak intensity
0 min	1.00
30 min	1.01
360 min	1.00

Stability of ETH in 2.5% MeOH aqueous solution was tested under conditions without light irradiation (Dark control experiments). A 2.5 mg/L ETH in Milli-Q water containing in 2.5% MeOH aqueous solution was stored in a quartz bottle for the photochemical experiments, and ETH was analyzed using LC after 30 min and 360 min. The relative peak intensities shown in Table S1 indicate the ratio between the peak areas at each time and the initial (0 min) peak area.

Both aqueous solutions were clear without precipitation. After storage of the ETH solution in the dark for 360 min, the LC analysis peak intensity did not change (Table S1) and no new peaks appeared. Therefore, no precipitation, adsorption, or degradation of ETH would occur during the experiment.

3. Appearance of the photodegradation equipment

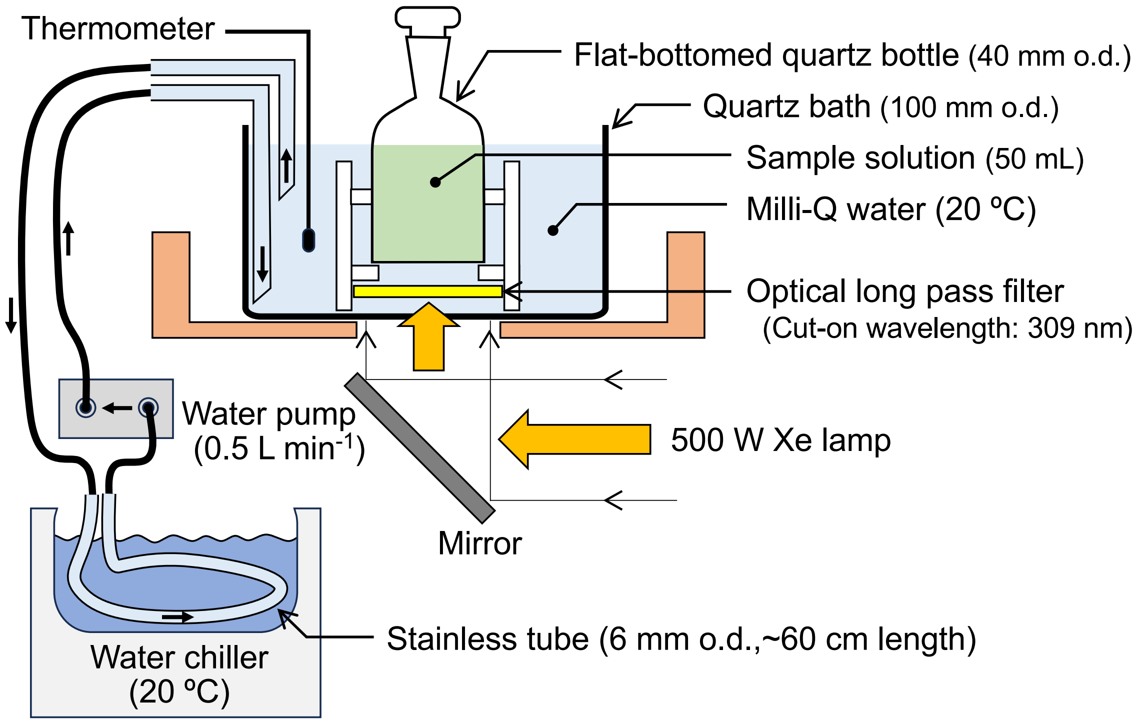

Fig. S2 (a) Schematic diagram of photochemical experimental system

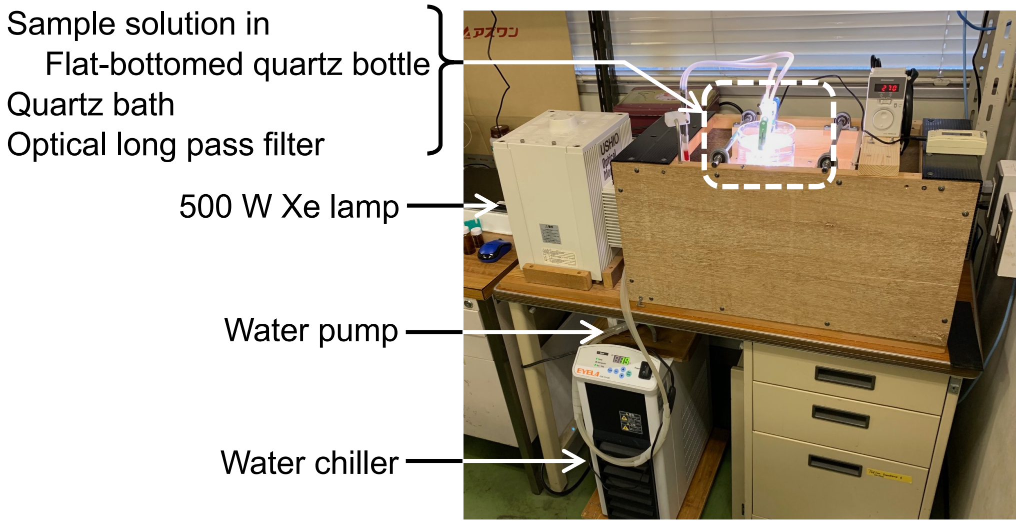

Fig. S2 (b) Picture of the photodegradation equipment

3. Results of LC analysis

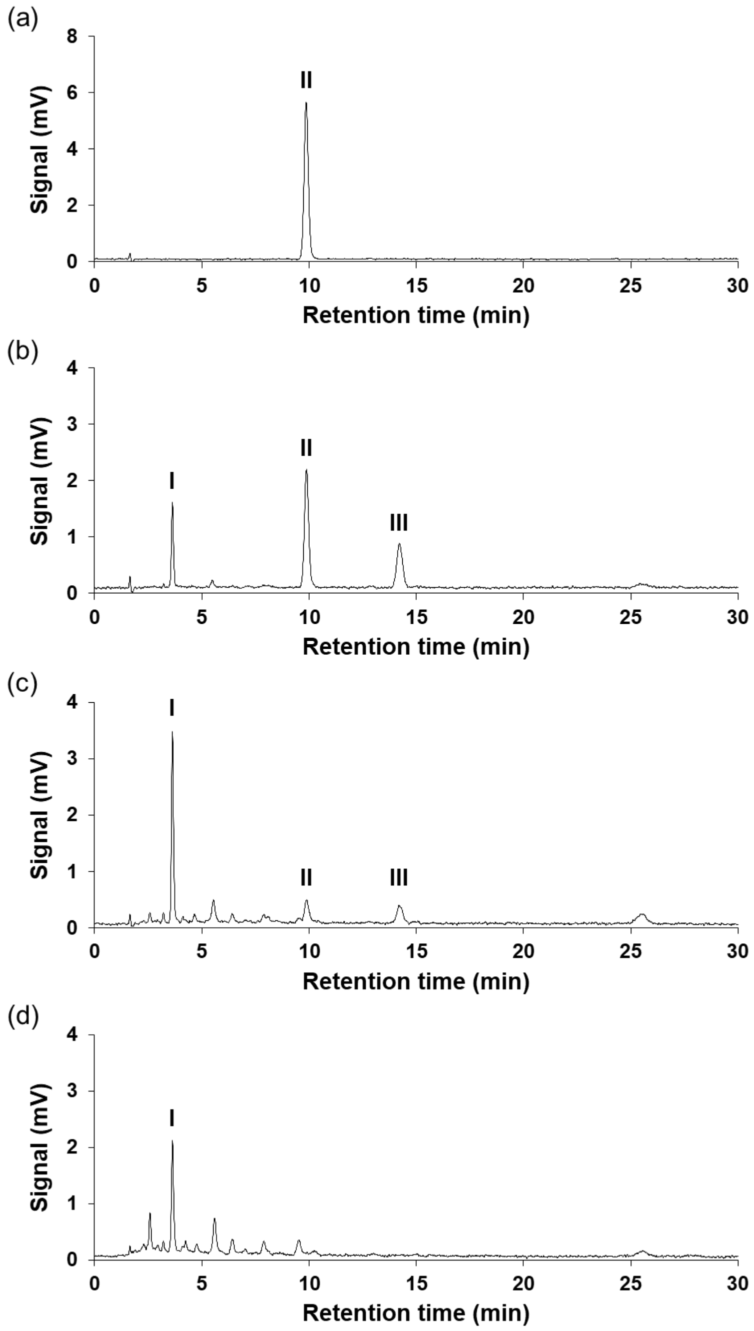

Fig. S3 UV chromatograms of aqueous solution of ETH containing 2.5% MeOH after (a) 0 min (before photoirradiation), (b) 30 min, (c) 90 min, and (d) 300 min photodegradation

The major peaks were identified as I : Benzimidazole of des-chloro-hydroxy-ETH, II : ETH, and III : Benzimidazole of ETH, respectively, based on the results of LC-MS/MS analysis (Table 1 and Fig. S4–5).

4. Results of LC-MS/MS analysis

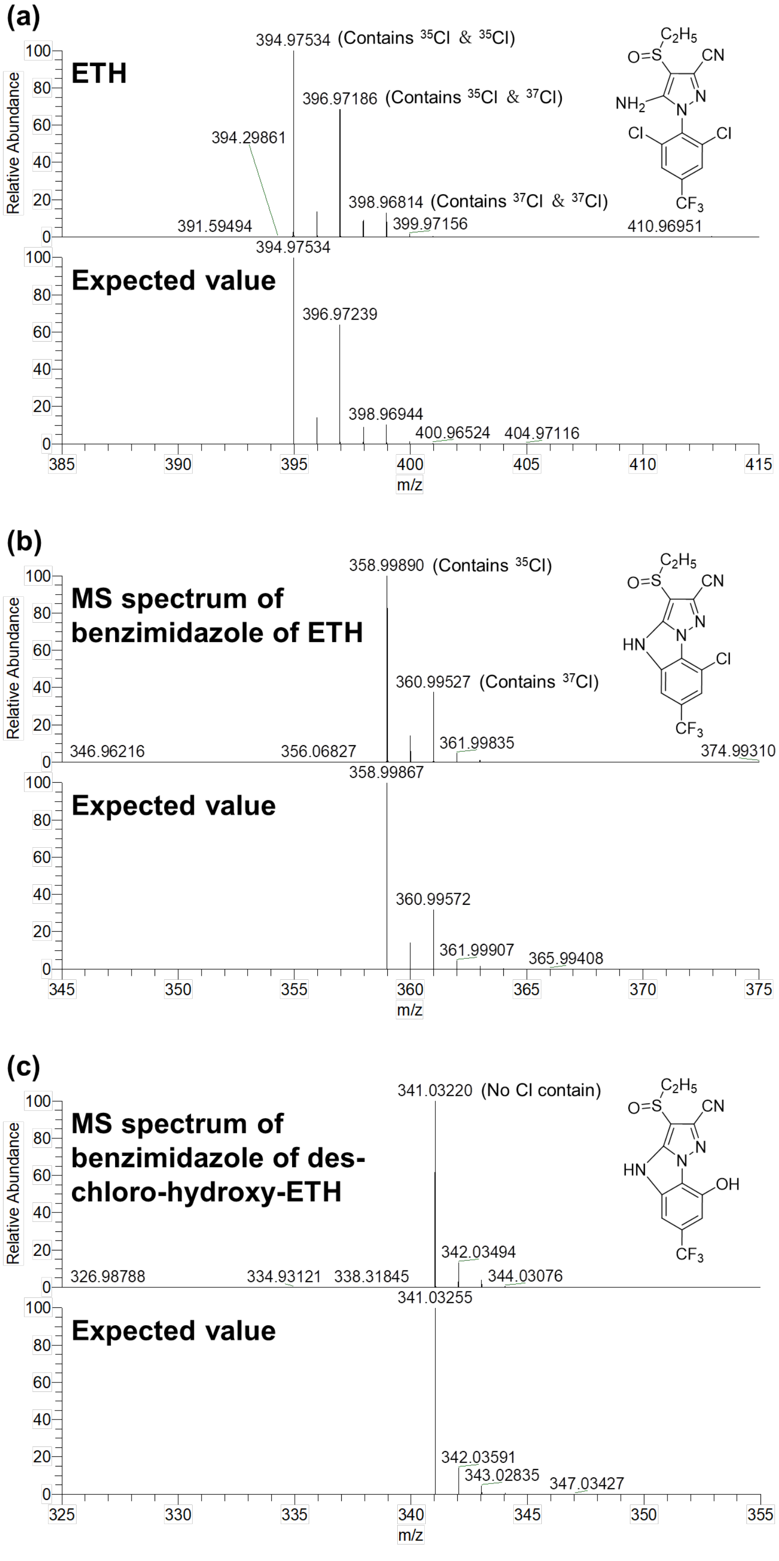

Fig. S4 Isotope pattern of (a) ETH, (b) Benzimidazole of ETH and (c) Benzimidazole of des-chloro-hydroxy-ETH in MS spectra

The main photodegradation products of ETH had a characteristic isotopic pattern that differed from that of ETH, a dichloride compound.

The ratio and mass difference of the major isotope peaks in benzimidazole of ETH were generally consistent with the two major isotopes of Cl (^{35}Cl [75.77%] and ^{37}Cl [24.23%]), and thus it was inferred to be a monochlorinated compound. On the other hand, no characteristic isotope peak attributed to chlorine was observed in benzimidazole of des-chloro-hydroxy-ETH, and it can be inferred that it is a no chlorinated compound.

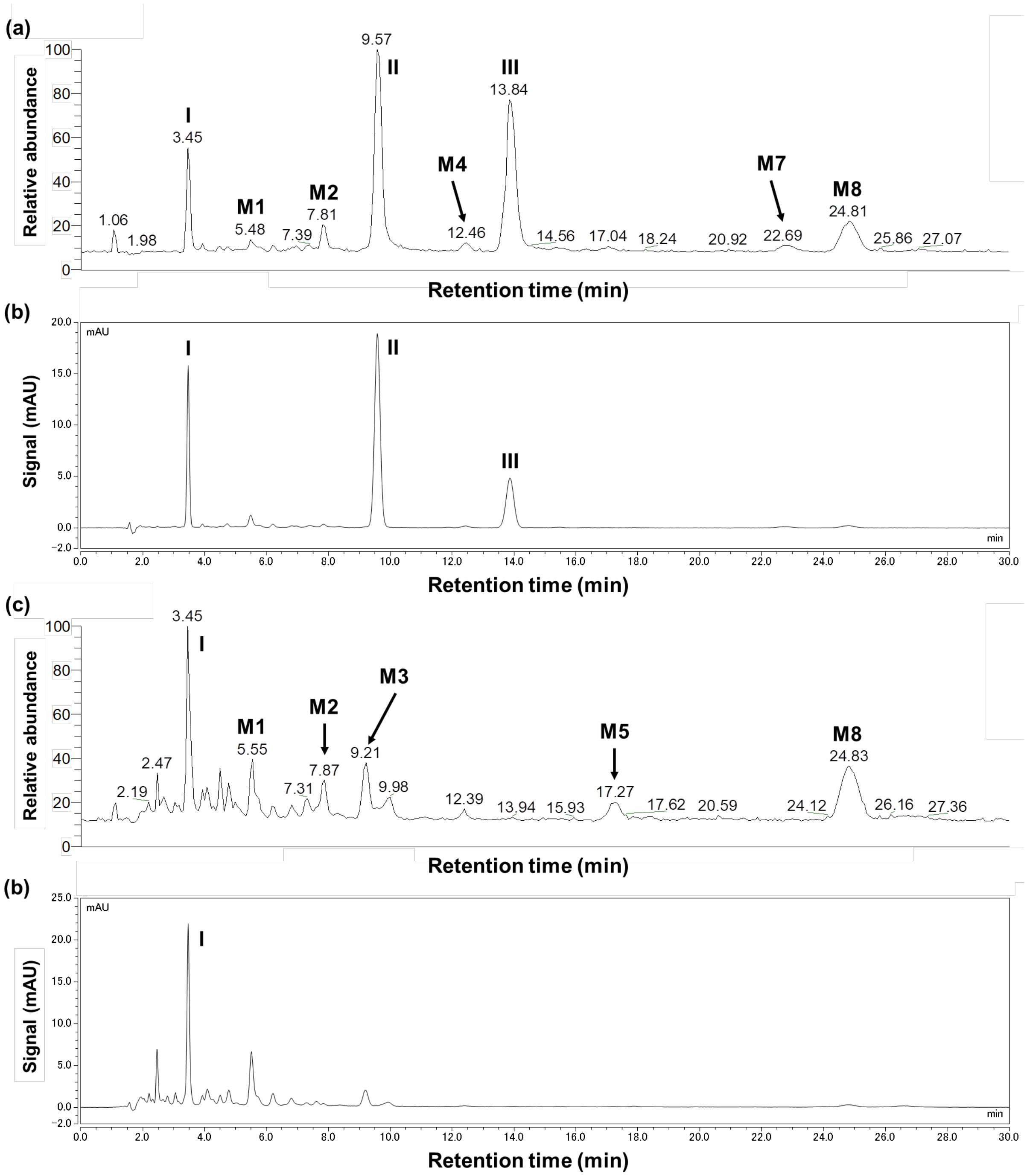

Fig. S5 The LC-MS total ion chromatogram (TIC) of photo-irradiated ETH solution after SPE concentration
(a) TIC of 30 min photo-irradiated solution after SPE concentration
(b) UV 280 nm chromatogram monitored simultaneously with (a)
(c) TIC of 300 min photo-irradiated solution after SPE concentration
(d) UV 280 nm chromatogram monitored simultaneously with (c)

In addition to peak I–III, many other peaks were appeared in the TIC. The *m/z* ratios in MS and MS/MS of these peaks were summarized in Table S2, and their chemical structures estimated were shown in Fig. 6.

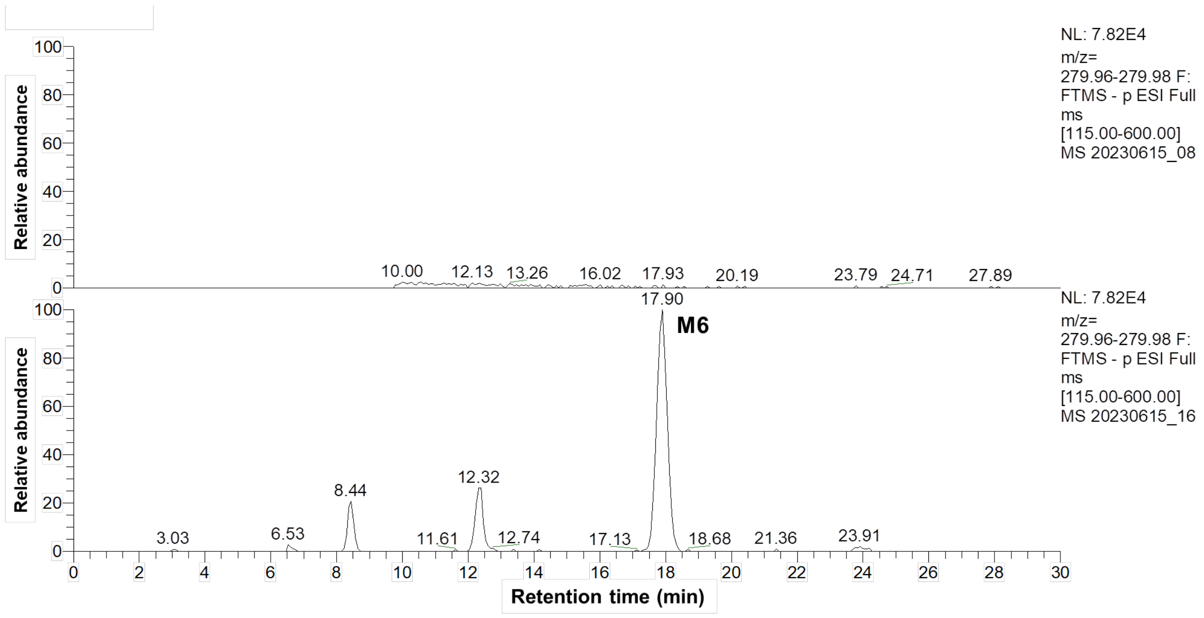

Fig. S6 The LC-MS extracted ion chromatogram (EIC) (Mass range: *m/z* ratios = 279.96–279.98) of photo-irradiated ETH solution after SPE concentration
Upper: EIC of 0 min photo-irradiated solution after SPE concentration
Lower: EIC of 300 min photo-irradiated solution after SPE concentration

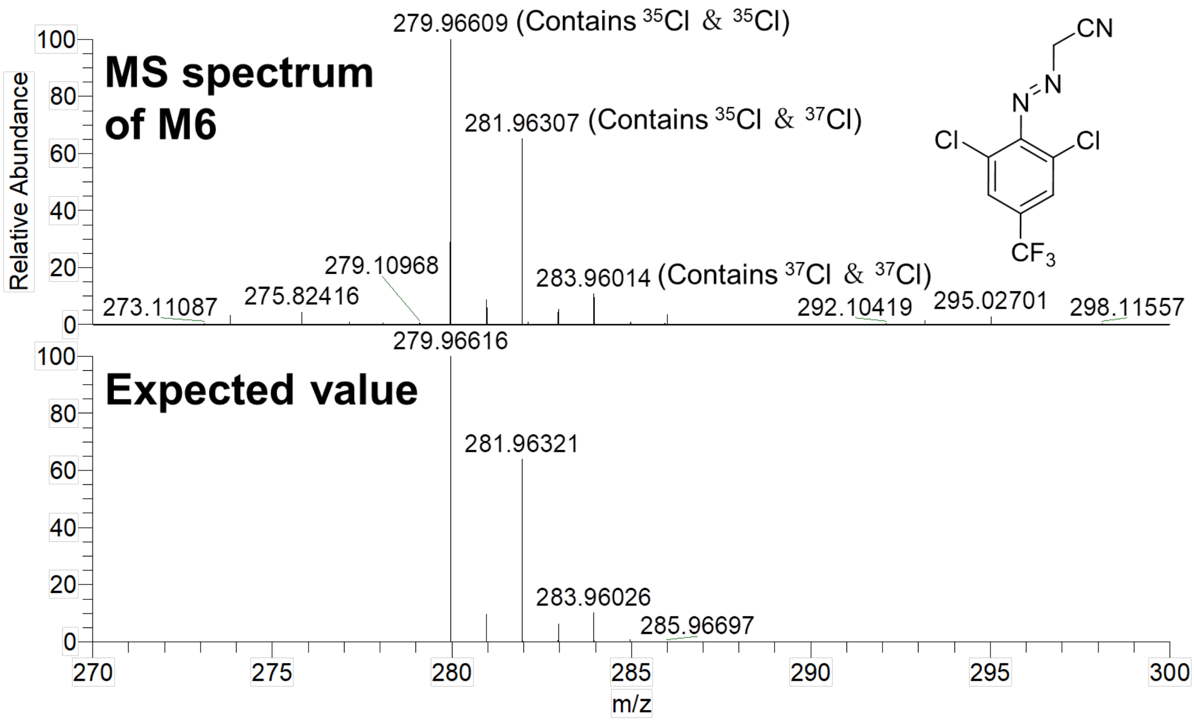

Fig. S7 Isotope pattern of MP6 in MS spectra

The MS spectrum of M6 confirmed the ratio and mass difference of the major isotope peaks characteristic of the dichloride compounds, as in ETH (Fig. S4).

Table S2 LC-MS/MS results of minor products M1–M8 corresponding to TIC or EIC shown in Fig. S5 and S6. The MS/MS (*m/z*) ratios are shown in order of peak intensity

Peak No.	MS (*m/z*)	MS/MS (*m/z*)	Reference
M1	262.98	219.0 (-CO_2)	JMPR 2018
M2	325.04	297.0 (-2H, -CN or -N-N)	This study
M3	325.04	296.0 (-H, -N-N or -C_2H_5)	This study
M4	410.97	375.1 (-H, -Cl)	USEPA 2011<br>FSCJ 2014<br>JMPR 2018<br>Chen et al. 2019
M5	325.04	296.0 (-H, -N-N or -C_2H_5), 305.1 (-H, -F)	This study
M6	279.97	244.0 (-H, -Cl), 252.0 (-2H, -CN)	Chen et al. 2019
M7	378.98	343.1 (-H, -Cl)	USEPA 2011<br>FSCJ 2014<br>JMPR 2018<br>Chen et al. 2019
M8	309.04	280.1 (-H, -N-N or -C_2H_5)	This study

6. Photodegradation and non-photochemical oxidation and reduction of ETH

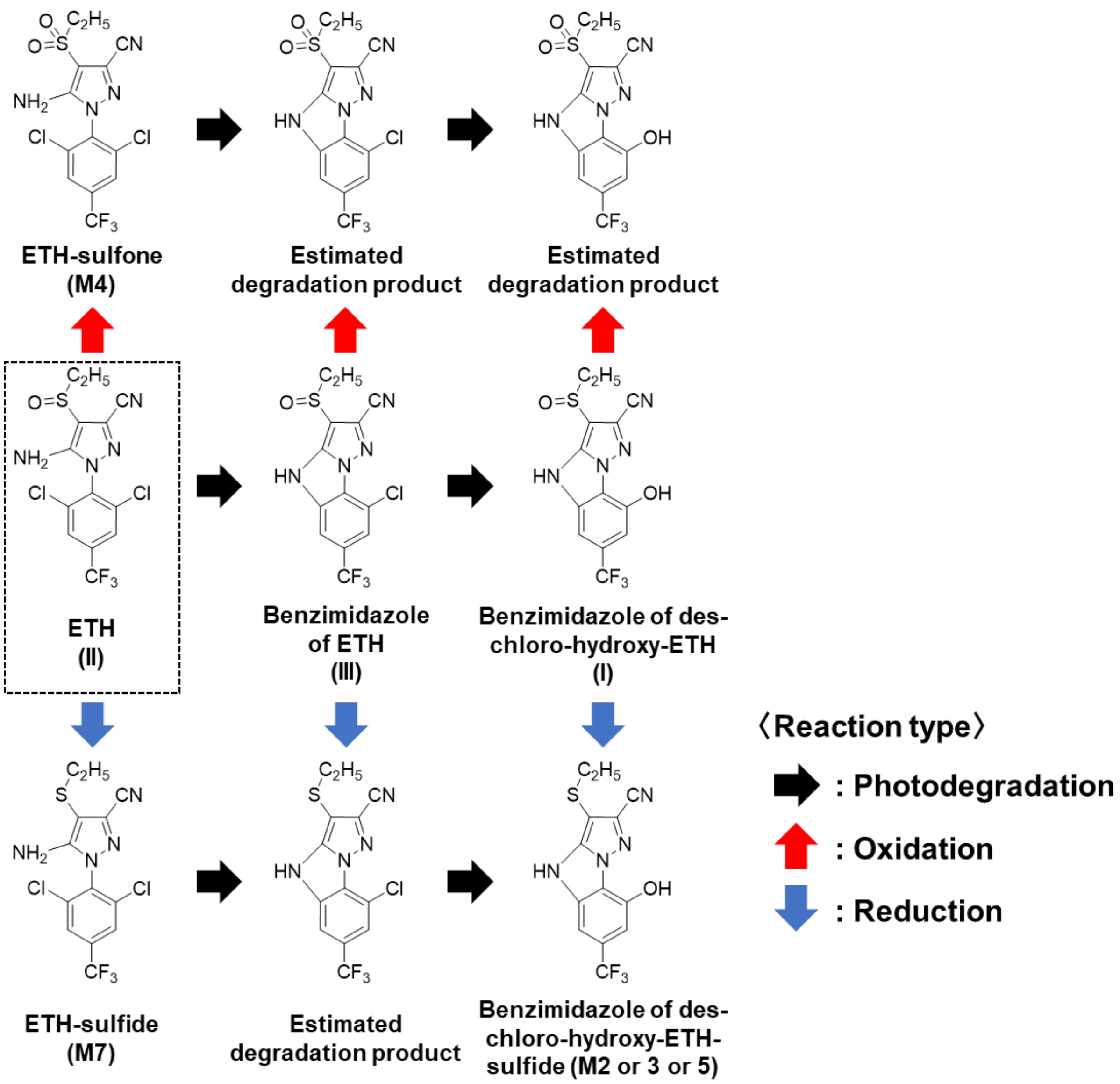

Fig. S8 Photodegradation and non-photochemical oxidation and reduction of ETH

ETH was photochromically degraded maintaining the sulfinyl structure. Therefore, the photochemical products of ETH may undergo oxidation and reduction of the sulfinyl structure in the dark, in natural environments similar to FIP and ETH. The reactions of ETH are complicated in natural environments.

END
